# Supplementary material for: Ontogenetic shifts in space use and habitat selection of tiger sharks (Galeocerdo cuvier) in The Bahamas
Source: PLoS One. 2025 Oct 30;20(10):e0335659. doi: 10.1371/journal.pone.0335659 (PMC12574918; doi:10.1371/journal.pone.0335659)
Supplement: S1 Fig — (DOCX) [file pone.0335659.s001.docx]

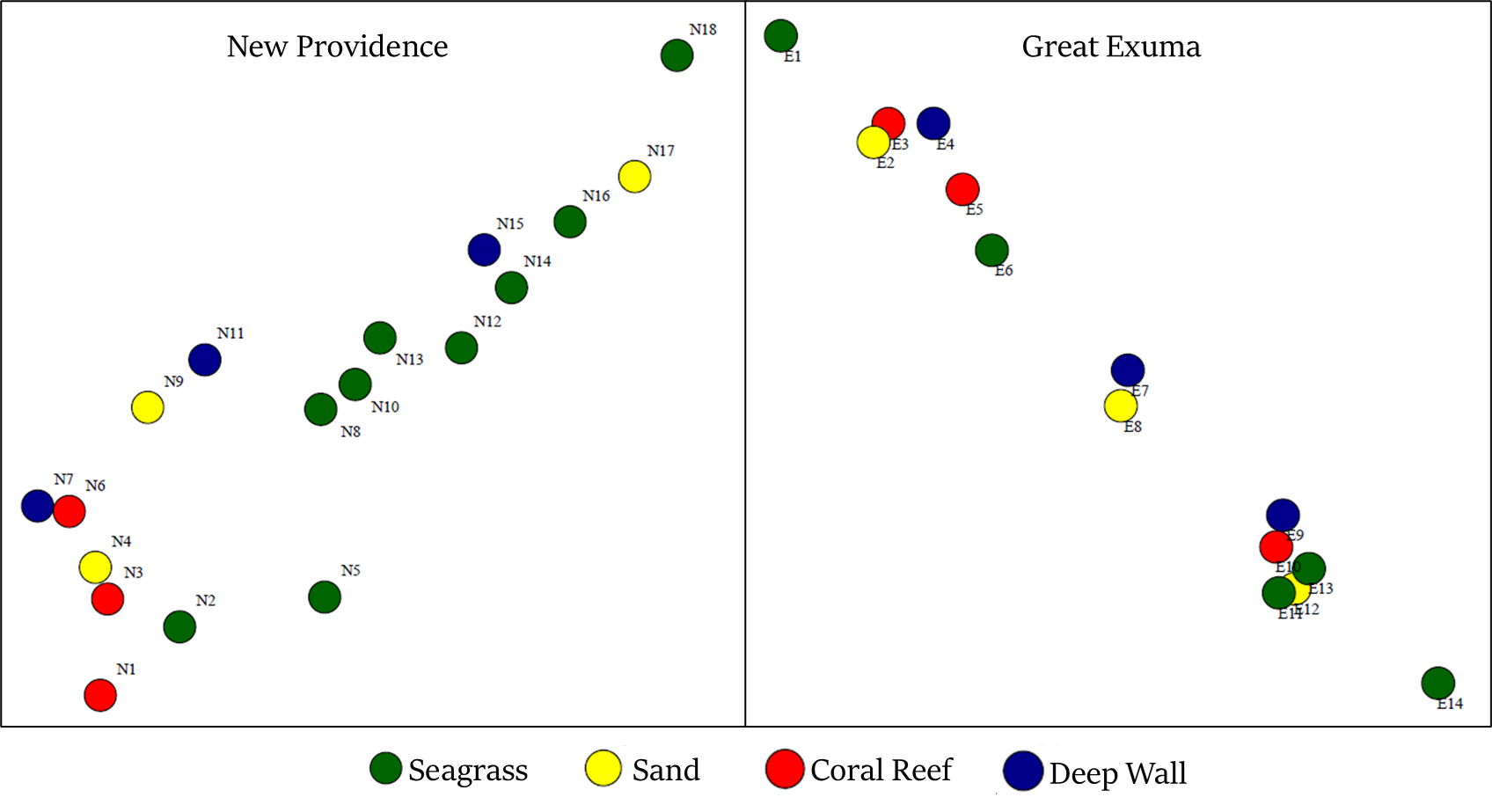


**S1 Fig.** Habitat type and location of acoustic receivers in New Providence and Great Exuma, The Bahamas.
