# Supplementary figures and images for: Ontogenetic shifts in space use and habitat selection of tiger sharks (Galeocerdo cuvier) in The Bahamas
Source: PLoS One. 2025 Oct 30;20(10):e0335659. doi: 10.1371/journal.pone.0335659 (PMC12574918; doi:10.1371/journal.pone.0335659)

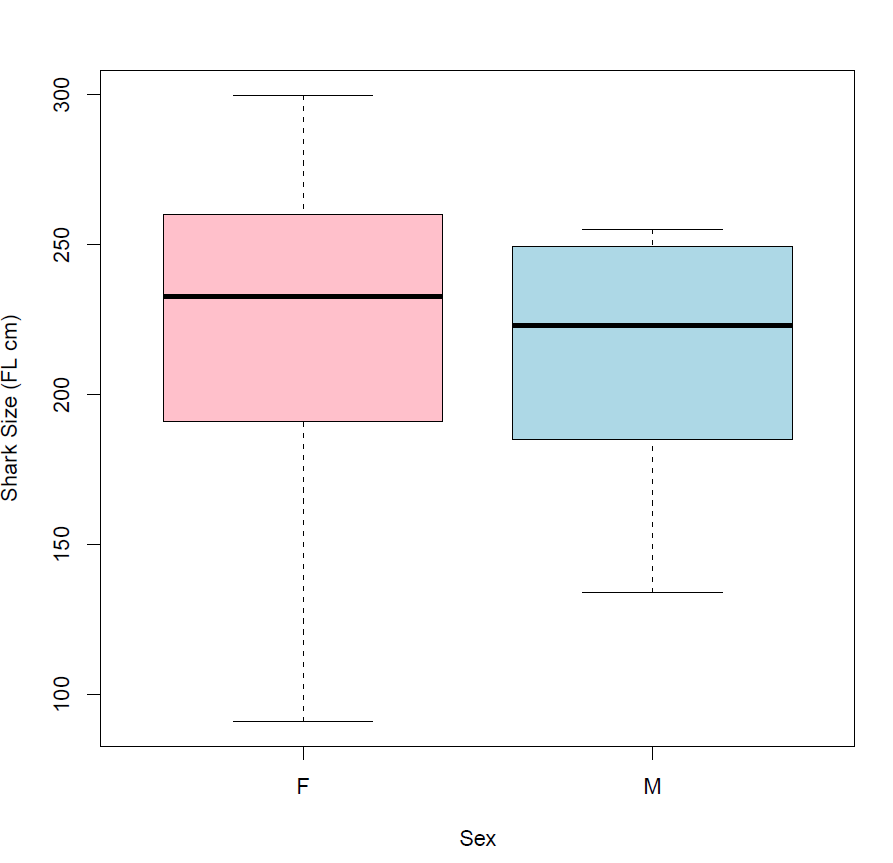


**S2 Fig.** Estimated fork length (cm) size at detection for female (n = 30) and male tiger sharks (n = 9).

Supplement: S2 Fig — (DOCX) [file pone.0335659.s002.docx]
