# Supplementary material for: Ontogenetic shifts in space use and habitat selection of tiger sharks (Galeocerdo cuvier) in The Bahamas
Source: PLoS One. 2025 Oct 30;20(10):e0335659. doi: 10.1371/journal.pone.0335659 (PMC12574918; doi:10.1371/journal.pone.0335659)
Supplement: S3 Fig — (DOCX) [file pone.0335659.s003.docx]

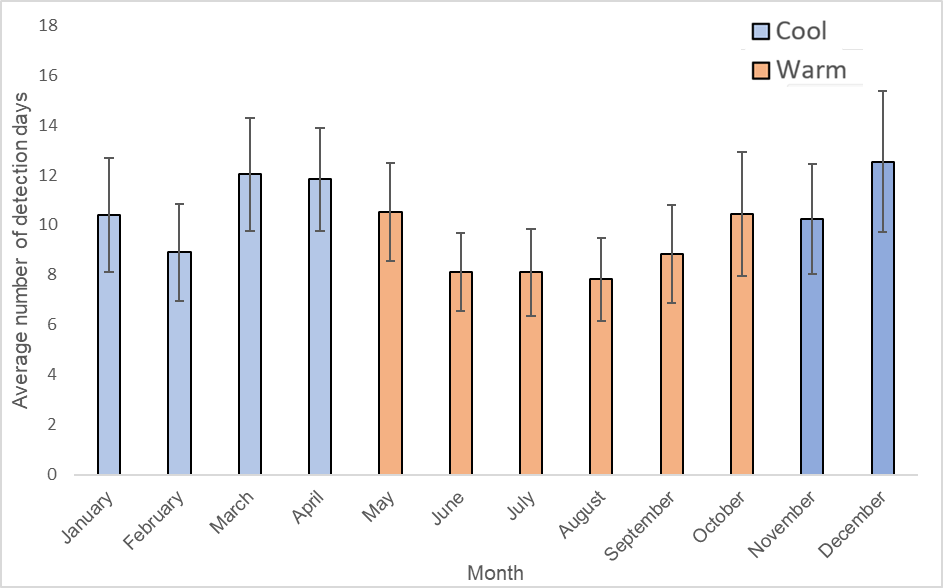


**S3 Fig.** Average number of detection days of acoustically detected tiger sharks (n = 39) in each month at New Providence and Great Exuma, The Bahamas and season (warm/cool).
