# Supplementary material for: Ontogenetic shifts in space use and habitat selection of tiger sharks (Galeocerdo cuvier) in The Bahamas
Source: PLoS One. 2025 Oct 30;20(10):e0335659. doi: 10.1371/journal.pone.0335659 (PMC12574918; doi:10.1371/journal.pone.0335659)
Supplement: S1 Table — (DOCX) [file pone.0335659.s004.docx]

**S1 Table**. Acoustic receiver station names, island, depth, habitat, code names and locations.

| Station | Island | Depth (m) | Habitat | Code | Latitude | Longitude | Install Date | Final Removal |
| --- | --- | --- | --- | --- | --- | --- | --- | --- |
| Southwest Reef | New Providence | 11 | Coral reef | N1 | 24.904 | -77.530 | 12/02/2022 | 04/01/2024 |
| Coral Harbor | New Providence | 5 | Seagrass | N2 | 24.944 | -77.440 | 09/11/2020 | 20/12/2023 |
| Reef HQ | New Providence | 8 | Coral reef | N3 | 24.961 | -77.522 | 02/10/2020 | 28/07/2022 |
| Bahama Mama | New Providence | 12 | Coral reef | N4 | 24.980 | -77.535 | 02/03/2021 | 17/09/2023 |
| Tiger Lane | New Providence | 3 | Seagrass | N5 | 24.962 | -77.277 | 20/11/2018 | 18/06/2021 |
| Goulding | New Providence | 12 | Coral reef | N6 | 25.013 | -77.565 | 02/05/2018 | 25/07/2022 |
| RD West | New Providence | 179 | Deep wall | N7 | 25.016 | -77.601 | 20/07/2020 | 27/07/2022 |
| Athol | New Providence | 2 | Seagrass | N8 | 25.065 | -77.276 | 16/11/2018 | 25/04/2022 |
| Kessel | New Providence | 6 | Coral reef | N9 | 25.074 | -77.476 | 09/05/2018 | 10/11/2021 |
| Payne Palace | New Providence | 6 | Seagrass | N10 | 25.079 | -77.237 | 16/11/2018 | 18/12/2023 |
| RD North | New Providence | 153 | Deep wall | N11 | 25.103 | -77.412 | 19/07/2020 | 28/07/2022 |
| South Rose | New Providence | 2 | Seagrass | N12 | 25.110 | -77.123 | 20/11/2018 | 20/03/2022 |
| North Rose | New Providence | 11 | Seagrass | N13 | 25.107 | -77.209 | 01/11/2020 | 05/01/2024 |
| Pierced Tongue | New Providence | 2 | Seagrass | N14 | 25.145 | -77.066 | 18/11/2018 | 22/12/2023 |
| RD East | New Providence | 181 | Deep wall | N15 | 25.168 | -77.097 | 22/07/2020 | 26/07/2022 |
| Link W | New Providence | 7 | Seagrass | N16 | 25.184 | -77.000 | 05/12/2020 | 26/03/2023 |
| Link M | New Providence | 7 | Sand | N17 | 25.211 | -76.927 | 04/12/2020 | 15/12/2023 |
| Link E | New Providence | 7 | Seagrass | N18 | 25.283 | -76.880 | 10/01/2021 | 24/10/2023 |
| Lee Stocking Island | Great Exuma | 6 | Seagrass | E1 | 23.783 | -76.113 | 21/03/2022 | 21/07/2022 |
| Inner Rat | Great Exuma | 9 | Sand | E2 | 23.735 | -76.056 | 19/11/2018 | 26/07/2023 |
| Outer Rat | Great Exuma | 13 | Coral reef | E3 | 23.744 | -76.047 | 28/03/2022 | 18/08/2022 |
| Deep Rat | Great Exuma | 207 | Deep wall | E4 | 23.744 | -76.019 | 05/08/2021 | 21/07/2022 |
| Three Sisters | Great Exuma | 12 | Coral reef | E5 | 23.715 | -76.001 | 06/05/2018 | 06/12/2020 |
| Piggy | Great Exuma | 5 | Seagrass | E6 | 23.688 | -75.983 | 22/03/2022 | 12/01/2024 |
| Deep Grand | Great Exuma | 181 | Deep wall | E7 | 23.635 | -75.899 | 04/08/2021 | 18/07/2022 |
| Emerald Bay | Great Exuma | 8 | Coral reef | E8 | 23.619 | -75.903 | 27/03/2022 | 25/01/2024 |
| Deep Georgetown | Great Exuma | 181 | Deep wall | E9 | 23.570 | -75.803 | 22/02/2019 | 21/07/2022 |
| Georgetown Pass | Great Exuma | 8 | Seagrass | E10 | 23.556 | -75.807 | 05/08/2021 | 12/04/2023 |
| Hoopers Bay | Great Exuma | 2 | Seagrass | E11 | 23.536 | -75.805 | 23/02/2020 | 09/01/2024 |
| Outer Hoopers | Great Exuma | 5 | Sand | E12 | 23.538 | -75.795 | 22/02/2019 | 24/01/2020 |
| Lily Cay | Great Exuma | 5 | Seagrass | E13 | 23.547 | -75.787 | 22/03/2022 | 13/01/2024 |
| Fowl Cay | Great Exuma | 5 | Coral reef | E14 | 23.496 | -75.707 | 23/02/2020 | 17/07/2022 |
