# Supplementary material for: Ontogenetic shifts in space use and habitat selection of tiger sharks (Galeocerdo cuvier) in The Bahamas
Source: PLoS One. 2025 Oct 30;20(10):e0335659. doi: 10.1371/journal.pone.0335659 (PMC12574918; doi:10.1371/journal.pone.0335659)
Supplement: S2 Table — Table modified from Wirsing et al. 2006. (DOCX) [file pone.0335659.s005.docx]

**S2 Table.** Growth rate estimates per shark size class based on Branstetter et al. 1987. Table modified from Wirsing et al. 2006.

| Size (cm FL) | 100 | 125 | 150 | 175 | 200 | 225 | 250 | 275 | 300 |
| --- | --- | --- | --- | --- | --- | --- | --- | --- | --- |
| Growth (cm FL year ^-1^) | 40 | 35 | 30 | 18 | 15 | 12 | 10 | 8 | 4 |
