# Supplementary material for: Ontogenetic shifts in space use and habitat selection of tiger sharks (Galeocerdo cuvier) in The Bahamas
Source: PLoS One. 2025 Oct 30;20(10):e0335659. doi: 10.1371/journal.pone.0335659 (PMC12574918; doi:10.1371/journal.pone.0335659)
Supplement: S3 Table — (DOCX) [file pone.0335659.s006.docx]

**S3 Table.** Attributes of tiger sharks that were detected on the acoustic receiver arrays.

| ID | Sex | TL (cm) | FL (cm) | FL (cm) at detection | Life-stage at tagging | Life-stage over study | Date Tagged | Estimated growth rate per year | Tagging Location | Latitude | Longitude |
| --- | --- | --- | --- | --- | --- | --- | --- | --- | --- | --- | --- |
| A69-1601-58346 | F | 298 | 250 | 266 | Sub-Adult | Adult | 06/02/2018 | 10 | The Exumas | 24.68 | -77.085 |
| A69-1602-26065 | F | 159 | 122 | 123 | Juvenile | Juvenile | 02/05/2018 | 40 | The Exumas | 23.564 | -75.806 |
| A69-1602-26071 | F | 290 | 240 | 267 | Sub-Adult | Adult | 02/05/2018 | 12 | The Exumas | 23.544 | -75.787 |
| A69-1602-26059 | F | 322 | 273 | 273 | Adult | Adult | 02/05/2018 | 10 | The Exumas | 23.531 | -75.779 |
| A69-1602-26066 | F | 304 | 248 | 248 | Sub-Adult | Sub-Adult | 03/05/2018 | 12 | The Exumas | 23.544 | -75.787 |
| A69-1602-26075 | F | 220 | 170 | 176 | Juvenile | Juvenile | 04/05/2018 | 30 | New Providence | 25.078 | -77.159 |
| A69-1602-26060 | F | 246 | 193 | 193 | Sub-Adult | Sub-Adult | 05/05/2018 | 18 | New Providence | 24.945 | -77.441 |
| A69-1602-24099 | F | 263 | 221 | 221 | Sub-Adult | Sub-Adult | 20/11/2018 | 15 | The Exumas | 24.743 | -76.857 |
| A69-1602-24098 | F | 322 | 239 | 239 | Sub-Adult | Sub-Adult | 20/02/2019 | 12 | The Exumas | 23.783 | -76.128 |
| A69-9006-5982 | F | 230 | 178 | 181 | Juvenile | Sub-Adult | 21/02/2019 | 18 | New Providence | 25.048 | -77.521 |
| A69-9006-5980 | F | 267 | 279 | 280 | Adult | Adult | 21/02/2019 | 8 | The Exumas | 23.564 | -75.806 |
| A69-1602-22693 | M | 276 | 239 | 255 | Sub-Adult | Adult | 21/02/2019 | 12 | The Exumas | 23.736 | -76.055 |
| A69-9006-5981 | M | 276 | 222 | 223 | Sub-Adult | Sub-Adult | 21/02/2019 | 15 | The Exumas | 23.718 | -76.005 |
| A69-1602-22692 | F | 230 | 190 | 191 | Sub-Adult | Sub-Adult | 22/02/2019 | 18 | New Providence | 25.081 | -77.193 |
| A69-9006-5983 | F | 230 | 190 | 191 | Sub-Adult | Sub-Adult | 22/02/2019 | 18 | New Providence | 24.951 | -77.479 |
| A69-1602-22689 | F | 304 | 247 | 265 | Sub-Adult | Adult | 24/05/2019 | 12 | New Providence | 25.108 | -77.157 |
| A69-1602-20239 | M | 167 | 132 | 134 | Juvenile | Juvenile | 30/05/2019 | 35 | The Exumas | 23.531 | -75.779 |
| A69-9001-4202 | F | 305 | 245 | 265 | Sub-Adult | Adult | 21/02/2020 | 12 | The Exumas | 23.718 | -76.022 |
| A69-9001-4204 | F | 270 | 225 | 226 | Sub-Adult | Sub-Adult | 23/02/2020 | 15 | The Exumas | 23.493 | -75.709 |
| A69-9001-4206 | F | 119 | 82 | 91 | YOY | YOY | 22/07/2020 | 40 | New Providence | 25.177 | -77.037 |
| A69-9001-4208 | F | 230 | 190 | 194 | Sub-Adult | Sub-Adult | 23/07/2020 | 18 | New Providence | 25.078 | -77.159 |
| A69-9001-64507 | F | 313 | 260 | 260 | Adult | Adult | 08/12/2020 | 10 | The Exumas | 23.564 | -75.806 |
| A69-9001-64504 | F | 186 | 147 | 147 | Juvenile | Juvenile | 13/12/2020 | 35 | The Exumas | 23.734 | -76.058 |
| A69-9001-2369 | F | 258 | 230 | 239 | Sub-Adult | Sub-Adult | 15/12/2020 | 12 | New Providence | 24.933 | -77.361 |
| A69-9001-2371 | F | 340 | 289 | 300 | Adult | Adult | 15/12/2020 | 8 | The Exumas | 23.531 | -75.779 |
| A69-9001-12031 | F | 212 | 162 | 164 | Juvenile | Juvenile | 03/08/2021 | 30 | The Exumas | 23.716 | -76.001 |
| A69-9001-12034 | F | 238 | 193 | 194 | Sub-Adult | Sub-Adult | 03/08/2021 | 18 | New Providence | 24.945 | -77.441 |
| A69-9001-8612 | M | 157 | 124 | 140 | Juvenile | Juvenile | 05/08/2021 | 40 | New Providence | 24.96 | -77.444 |
| A69-9001-63269 | F | 303 | 252 | 252 | Sub-Adult | Sub-Adult | 12/08/2021 | 10 | The Exumas | 23.73 | -76.026 |
| A69-9001-60472 | F | 261 | 225 | 225 | Sub-Adult | Sub-Adult | 19/11/2021 | 15 | New Providence | 24.933 | -77.361 |
| A69-9001-60475 | M | 306 | 249 | 249 | Sub-Adult | Sub-Adult | 20/11/2021 | 12 | The Exumas | 23.501 | -75.723 |
| A69-9001-60481 | M | 225 | 181 | 185 | Juvenile | Sub-Adult | 14/12/2021 | 18 | New Providence | 25.078 | -77.159 |
| A69-9001-60507 | F | 288 | 239 | 241 | Sub-Adult | Sub-Adult | 14/12/2021 | 12 | New Providence | 25.084 | -77.231 |
| A69-9001-60487 | M | 305 | 253 | 253 | Sub-Adult | Adult | 15/12/2021 | 10 | New Providence | 25.021 | -77.554 |
| A69-9001-60476 | M | 300 | 243 | 244 | Sub-Adult | Sub-Adult | 16/12/2021 | 12 | The Exumas | 23.538 | -75.786 |
| A69-9001-60477 | F | 266 | 243 | 243 | Sub-Adult | Sub-Adult | 08/02/2022 | 12 | The Exumas | 23.493 | -75.709 |
| A69-9001-60495 | F | 276 | 239 | 240 | Sub-Adult | Sub-Adult | 23/03/2022 | 12 | The Exumas | 23.505 | -75.741 |
| A69-9001-60503 | M | 242 | 195 | 195 | Sub-Adult | Sub-Adult | 28/03/2022 | 18 | The Exumas | 24.743 | -76.857 |
| A69-9001-60505 | F | 178 | 135 | 138 | Juvenile | Juvenile | 30/05/2022 | 35 | The Exumas | 23.531 | -75.779 |
