# Supplementary material for: Ontogenetic shifts in space use and habitat selection of tiger sharks (Galeocerdo cuvier) in The Bahamas
Source: PLoS One. 2025 Oct 30;20(10):e0335659. doi: 10.1371/journal.pone.0335659 (PMC12574918; doi:10.1371/journal.pone.0335659)
Supplement: Attachment — Submitted filename: Response.docx [file pone.0335659.s008.docx]

PONE-D-25-14869

Space use and habitat selection of tiger sharks (Galeocerdo cuvier) across ontogeny throughout The Bahamas

PLOS ONE

Dear Dr. Ayres,

Thank you for submitting your manuscript to PLOS ONE. After careful consideration, we feel that it has merit but does not fully meet PLOS ONE’s publication criteria as it currently stands. Therefore, we invite you to submit a revised version of the manuscript that addresses the points raised during the review process.

Both reviewers saw merit in the study, and have provided sensible recommendations for how the quality of the manuscript might be improved. Please bear these comments in mind when revising the manuscript.

Please submit your revised manuscript by Aug 29 2025 11:59PM. If you will need more time than this to complete your revisions, please reply to this message or contact the journal office at [plosone@plos.org](mailto:plosone@plos.org). When you're ready to submit your revision, log on to https://www.editorialmanager.com/pone/ and select the 'Submissions Needing Revision' folder to locate your manuscript file.

Please include the following items when submitting your revised manuscript:

- A rebuttal letter that responds to each point raised by the academic editor and reviewer(s). You should upload this letter as a separate file labeled 'Response to Reviewers'.
- A marked-up copy of your manuscript that highlights changes made to the original version. You should upload this as a separate file labeled 'Revised Manuscript with Track Changes'.
- An unmarked version of your revised paper without tracked changes. You should upload this as a separate file labeled 'Manuscript'.

If you would like to make changes to your financial disclosure, please include your updated statement in your cover letter. Guidelines for resubmitting your figure files are available below the reviewer comments at the end of this letter.

If applicable, we recommend that you deposit your laboratory protocols in protocols.io to enhance the reproducibility of your results. Protocols.io assigns your protocol its own identifier (DOI) so that it can be cited independently in the future. For instructions see: [https://journals.plos.org/plosone/s/submission-guidelines#loc-laboratory-protocols](https://na01.safelinks.protection.outlook.com/?url=https%3A%2F%2Ftrack.editorialmanager.com%2FCL0%2Fhttps%3A%252F%252Fjournals.plos.org%252Fplosone%252Fs%252Fsubmission-guidelines%2523loc-laboratory-protocols%2F1%2F010f01980e13896c-197f2c47-38d3-4968-bb77-203727948264-000000%2FpCtAW4rWPjTC2hj_7n_PnjRT4UpbuasMoScX878Znxc%3D219&data=05%7C02%7C%7C104aeca9ad1d4b4b992208ddc39bd33d%7C84df9e7fe9f640afb435aaaaaaaaaaaa%7C1%7C0%7C638881796209541321%7CUnknown%7CTWFpbGZsb3d8eyJFbXB0eU1hcGkiOnRydWUsIlYiOiIwLjAuMDAwMCIsIlAiOiJXaW4zMiIsIkFOIjoiTWFpbCIsIldUIjoyfQ%3D%3D%7C0%7C%7C%7C&sdata=8ysXkZz3ZVwgTeyzJT%2FvkNgadQIvvSl2ZaN5JEa76NQ%3D&reserved=0). Additionally, PLOS ONE offers an option for publishing peer-reviewed Lab Protocol articles, which describe protocols hosted on protocols.io. Read more information on sharing protocols at [https://plos.org/protocols?utm_medium=editorial-email&utm_source=authorletters&utm_campaign=protocols](https://na01.safelinks.protection.outlook.com/?url=https%3A%2F%2Ftrack.editorialmanager.com%2FCL0%2Fhttps%3A%252F%252Fplos.org%252Fprotocols%253Futm_medium%3Deditorial-email%2526utm_source%3Dauthorletters%2526utm_campaign%3Dprotocols%2F1%2F010f01980e13896c-197f2c47-38d3-4968-bb77-203727948264-000000%2FWoVF45gmsCBHzSMbGpRWmaRX_ue_pQYhOORJjHcEUBQ%3D219&data=05%7C02%7C%7C104aeca9ad1d4b4b992208ddc39bd33d%7C84df9e7fe9f640afb435aaaaaaaaaaaa%7C1%7C0%7C638881796209575738%7CUnknown%7CTWFpbGZsb3d8eyJFbXB0eU1hcGkiOnRydWUsIlYiOiIwLjAuMDAwMCIsIlAiOiJXaW4zMiIsIkFOIjoiTWFpbCIsIldUIjoyfQ%3D%3D%7C0%7C%7C%7C&sdata=D3O3lRv05NFwvWgl2%2BSW7Cee%2BJDuQ95uYO62zWqEfbQ%3D&reserved=0).

We look forward to receiving your revised manuscript.

Kind regards,

Joel Harrison Gayford
Academic Editor
PLOS ONE

Journal Requirements:

When submitting your revision, we need you to address these additional requirements.

1. Please ensure that your manuscript meets PLOS ONE's style requirements, including those for file naming. The PLOS ONE style templates can be found at

https://journals.plos.org/plosone/s/file?id=wjVg/PLOSOne_formatting_sample_main_body.pdf and

[https://journals.plos.org/plosone/s/file?id=ba62/PLOSOne_formatting_sample_title_authors_affiliations.pdf](https://na01.safelinks.protection.outlook.com/?url=https%3A%2F%2Ftrack.editorialmanager.com%2FCL0%2Fhttps%3A%252F%252Fjournals.plos.org%252Fplosone%252Fs%252Ffile%253Fid%3Dba62%252FPLOSOne_formatting_sample_title_authors_affiliations.pdf%2F1%2F010f01980e13896c-197f2c47-38d3-4968-bb77-203727948264-000000%2FDlucpFtefzam4xK0Y_mK5NIlRPiajtlMzng-amro9Ro%3D219&data=05%7C02%7C%7C104aeca9ad1d4b4b992208ddc39bd33d%7C84df9e7fe9f640afb435aaaaaaaaaaaa%7C1%7C0%7C638881796209594217%7CUnknown%7CTWFpbGZsb3d8eyJFbXB0eU1hcGkiOnRydWUsIlYiOiIwLjAuMDAwMCIsIlAiOiJXaW4zMiIsIkFOIjoiTWFpbCIsIldUIjoyfQ%3D%3D%7C0%7C%7C%7C&sdata=NOTHP8zlX7yrWf6apngm%2FGFczr2KKTFDm0NMUAsjRnE%3D&reserved=0)

***Response: Style changed to match requirements in pdf.***

2. We note that the grant information you provided in the ‘Funding Information’ and ‘Financial Disclosure’ sections do not match.

When you resubmit, please ensure that you provide the correct grant numbers for the awards you received for your study in the ‘Funding Information’ section.

***Response – funding removed from the manuscript***

3. Thank you for stating the following in the Acknowledgments Section of your manuscript:
We thank our Bahamian partners and stakeholders who have enabled and supported this work: Gittens and the Dept.Marine Resources, E. Carey and S. Cant from Bahamas National Trust, S.Cove from Stuart Cove’s, J. Todd, P. Nicholson, R. Sands from Grand Isle Resort, A. Phillips and A. Musgrove from Bahamas Dive Guides, Dive Exuma, and The Exuma Foundation. We are particularly grateful to the following partners for logistical and operational support: Stuart Cove’s, The International Seakeepers Association, The Grand Isle Resort, GIR Bahamas, M/YMarcato, J. and M. McClurg, Fleet Miami, R/V Sharkwater, Atlantis, Bahamas Dive Guides, Dive Exuma, The Exuma Foundation, Vemco, and the Ocean Tracking Network. For field work support, we thank B. Shea, O. Dixon, S. Aldridge, J. Halvorsen, E. Staaterman, E. Quintero, J. Sternlicht, T. Gilbert, J. Roth, S. Moorhead, E. Sudal, and M. Adunni. This work was covered under a permit to Austin Gallagher from the Department of Marine Resources. We also thank our funders: The Wanderlust Fund, The King Family, Sternlicht Family Foundation, Lush, Maverick1000, National Geographic Wild, Seaworld and Busch Gardens Conservation Fund, Thayer Academy, WCPD Foundation, and Anomaly Entertainment.

We note that you have provided funding information that is not currently declared in your Funding Statement. However, funding information should not appear in the Acknowledgments section or other areas of your manuscript. We will only publish funding information present in the Funding Statement section of the online submission form.
Please remove any funding-related text from the manuscript and let us know how you would like to update your Funding Statement. Currently, your Funding Statement reads as follows:
This work was supported by grants to BTW from the following funders: The Wanderlust Fund, The King Family, Sternlicht Family Foundation, Lush, Maverick1000, National Geographic Wild, Seaworld and Busch Gardens Conservation Fund, Thayer Academy, WCPD Foundation, and Anomaly Entertainment. The funders did not play any role in the study design, data collection and analysis, decision to publish, or preparation of the manuscript

**Response: Removed from manuscript**

Please include your amended statements within your cover letter; we will change the online submission form on your behalf.

***Response: This is the funding information: This work was supported by grants to BTW from the following funders: The Wanderlust Fund, The King Family, Sternlicht Family Foundation, Lush, Maverick1000, National Geographic Wild, Seaworld and Busch Gardens Conservation Fund, Thayer Academy, WCPD Foundation, and Anomaly Entertainment. These funders were not involved in the study design, collection, analysis, interpretation of data, the writing of this article or the decision to submit it for publication.***

4. Your ethics statement should only appear in the Methods section of your manuscript. If your ethics statement is written in any section besides the Methods, please delete it from any other section.

**Response: Only in Method section**

5. In the online submission form, you indicated that the raw data supporting the conclusions of this article will be made available by the authors upon request.

All PLOS journals now require all data underlying the findings described in their manuscript to be freely available to other researchers, either a. In a public repository, b. Within the manuscript itself, or c. Uploaded as supplementary information.
This policy applies to all data except where public deposition would breach compliance with the protocol approved by your research ethics board. If your data cannot be made publicly available for ethical or legal reasons (e.g., public availability would compromise patient privacy), please explain your reasons on resubmission and your exemption request will be escalated for approval.

**Response: Data will be made publicly available**

6. When completing the data availability statement of the submission form, you indicated that you will make your data available on acceptance. We strongly recommend all authors decide on a data sharing plan before acceptance, as the process can be lengthy and hold up publication timelines. Please note that, though access restrictions are acceptable now, your entire data will need to be made freely accessible if your manuscript is accepted for publication. This policy applies to all data except where public deposition would breach compliance with the protocol approved by your research ethics board. If you are unable to adhere to our open data policy, please kindly revise your statement to explain your reasoning and we will seek the editor's input on an exemption. Please be assured that, once you have provided your new statement, the assessment of your exemption will not hold up the peer review process.

**Response: Entire data will be made freely accessible**

7. We note that Figures 1 and 7 in your submission contain map/satellite images which may be copyrighted. All PLOS content is published under the Creative Commons Attribution License (CC BY 4.0), which means that the manuscript, images, and Supporting Information files will be freely available online, and any third party is permitted to access, download, copy, distribute, and use these materials in any way, even commercially, with proper attribution. For these reasons, we cannot publish previously copyrighted maps or satellite images created using proprietary data, such as Google software (Google Maps, Street View, and Earth). For more information, see our copyright guidelines: http://journals.plos.org/plosone/s/licenses-and-copyright.

We require you to either (a) present written permission from the copyright holder to publish these figures specifically under the CC BY 4.0 license, or (b) remove the figures from your submission:

a. You may seek permission from the original copyright holder of Figures 1 and 7 to publish the content specifically under the CC BY 4.0 license.

We recommend that you contact the original copyright holder with the Content Permission Form (http://journals.plos.org/plosone/s/file?id=7c09/content-permission-form.pdf) and the following text:

“I request permission for the open-access journal PLOS ONE to publish XXX under the Creative Commons Attribution License (CCAL) CC BY 4.0 (http://creativecommons.org/licenses/by/4.0/). Please be aware that this license allows unrestricted use and distribution, even commercially, by third parties. Please reply and provide explicit written permission to publish XXX under a CC BY license and complete the attached form.”

Please upload the completed Content Permission Form or other proof of granted permissions as an ""Other"" file with your submission.

In the figure caption of the copyrighted figure, please include the following text: “Reprinted from [ref] under a CC BY license, with permission from [name of publisher], original copyright [original copyright year].”

b. If you are unable to obtain permission from the original copyright holder to publish these figures under the CC BY 4.0 license or if the copyright holder’s requirements are incompatible with the CC BY 4.0 license, please either i) remove the figure or ii) supply a replacement figure that complies with the CC BY 4.0 license. Please check copyright information on all replacement figures and update the figure caption with source information. If applicable, please specify in the figure caption text when a figure is similar but not identical to the original image and is therefore for illustrative purposes only.

The following resources for replacing copyrighted map figures may be helpful:

USGS National Map Viewer (public domain): http://viewer.nationalmap.gov/viewer/

The Gateway to Astronaut Photography of Earth (public domain): http://eol.jsc.nasa.gov/sseop/clickmap/

Maps at the CIA (public domain): https://www.cia.gov/library/publications/the-world-factbook/index.html and https://www.cia.gov/library/publications/cia-maps-publications/index.html

NASA Earth Observatory (public domain): http://earthobservatory.nasa.gov/

Landsat: http://landsat.visibleearth.nasa.gov/

USGS EROS (Earth Resources Observatory and Science (EROS) Center) (public domain): http://eros.usgs.gov/#

Natural Earth (public domain): [http://www.naturalearthdata.com/](https://na01.safelinks.protection.outlook.com/?url=http%3A%2F%2Ftrack.editorialmanager.com%2FCL0%2Fhttp%3A%252F%252Fwww.naturalearthdata.com%252F%2F1%2F010f01980e13896c-197f2c47-38d3-4968-bb77-203727948264-000000%2F3lENdkYEkCts_-j23EiNQ3_-Czr-DXTmsGn_0U0aGpU%3D219&data=05%7C02%7C%7C104aeca9ad1d4b4b992208ddc39bd33d%7C84df9e7fe9f640afb435aaaaaaaaaaaa%7C1%7C0%7C638881796209607865%7CUnknown%7CTWFpbGZsb3d8eyJFbXB0eU1hcGkiOnRydWUsIlYiOiIwLjAuMDAwMCIsIlAiOiJXaW4zMiIsIkFOIjoiTWFpbCIsIldUIjoyfQ%3D%3D%7C0%7C%7C%7C&sdata=1B9%2FU6MGpNv2LXeiXo62n9Tb3ZkHXgRHO3NTuUCt0xs%3D&reserved=0)

**Response: Figure 1: We created the map in ArcGis.** **Source of world map layer: Esri, Maxar, Earthstar Geographics, and the GIS User Community, “World Imagery’ (basemap) Scale not given. February 19th 2012 https://www.arcgis.com/home/item.html?id=10df2279f9684e4a9f6a7f08febac2a9 (Accessed 7th January 2025).**

**Figure 7. The map was generated in R using the rnaturalearth (South, 2023) and ggplot2 (Wickham, 2016) packages with vector data from Natural Earth (version 4.1.0, 2023) - public domain.**

8. Please include captions for your Supporting Information files at the end of your manuscript, and update any in-text citations to match accordingly. Please see our Supporting Information guidelines for more information: http://journals.plos.org/plosone/s/supporting-information.

**Response: Captions and citations included.**

9. If the reviewer comments include a recommendation to cite specific previously published works, please review and evaluate these publications to determine whether they are relevant and should be cited. There is no requirement to cite these works unless the editor has indicated otherwise. 
[Note: HTML markup is below. Please do not edit.]

Reviewers' comments:

Reviewer's Responses to Questions

Comments to the Author

1. Is the manuscript technically sound, and do the data support the conclusions?

The manuscript must describe a technically sound piece of scientific research with data that supports the conclusions. Experiments must have been conducted rigorously, with appropriate controls, replication, and sample sizes. The conclusions must be drawn appropriately based on the data presented.

Reviewer #1: Partly

Reviewer #2: Yes

2. Has the statistical analysis been performed appropriately and rigorously?

Reviewer #1: Yes

Reviewer #2: Yes

3. Have the authors made all data underlying the findings in their manuscript fully available?

The [PLOS Data policy](https://na01.safelinks.protection.outlook.com/?url=http%3A%2F%2Ftrack.editorialmanager.com%2FCL0%2Fhttp%3A%252F%252Fwww.plosone.org%252Fstatic%252Fpolicies.action%2523sharing%2F1%2F010f01980e13896c-197f2c47-38d3-4968-bb77-203727948264-000000%2F7UD54v04LGAg7x96Cm3mMNcfrSeZH8YGDqeSAQB4jko%3D219&data=05%7C02%7C%7C104aeca9ad1d4b4b992208ddc39bd33d%7C84df9e7fe9f640afb435aaaaaaaaaaaa%7C1%7C0%7C638881796209622011%7CUnknown%7CTWFpbGZsb3d8eyJFbXB0eU1hcGkiOnRydWUsIlYiOiIwLjAuMDAwMCIsIlAiOiJXaW4zMiIsIkFOIjoiTWFpbCIsIldUIjoyfQ%3D%3D%7C0%7C%7C%7C&sdata=PRZUnSnbEB%2Bihf9zprlFJTJfJF2FoPe%2Fa4lrR5wO5nc%3D&reserved=0) requires authors to make all data underlying the findings described in their manuscript fully available without restriction, with rare exception (please refer to the Data Availability Statement in the manuscript PDF file). The data should be provided as part of the manuscript or its supporting information, or deposited to a public repository. For example, in addition to summary statistics, the data points behind means, medians and variance measures should be available. If there are restrictions on publicly sharing data—e.g. participant privacy or use of data from a third party—those must be specified.

Reviewer #1: Yes

Reviewer #2: Yes

4. Is the manuscript presented in an intelligible fashion and written in standard English?

PLOS ONE does not copyedit accepted manuscripts, so the language in submitted articles must be clear, correct, and unambiguous. Any typographical or grammatical errors should be corrected at revision, so please note any specific errors here.

Reviewer #1: No

Reviewer #2: Yes

5. Review Comments to the Author

Please use the space provided to explain your answers to the questions above. You may also include additional comments for the author, including concerns about dual publication, research ethics, or publication ethics. (Please upload your review as an attachment if it exceeds 20,000 characters)

Reviewer #1: This manuscript titled "Space use and habitat selection of tiger sharks (Galeocerdo cuvier) across ontogeny throughout The Bahamas" (PONE-D-25-14869) presents an analysis of tiger shark spatial ecology using a five-year acoustic telemetry dataset from The Bahamas. The study is in a good position to advance our understanding of ontogenetic shifts in habitat use and regional connectivity of an ecologically important apex predator in a vulnerable subtropical ecosystem. The combination of network analysis, GAM modeling, and integration with the Ocean Tracking Network makes this potentially a strong and highly relevant contribution. However, improvements in organization, writing clarity, better contextualizing of the literature in the Introduction and Discussion would significantly strengthen the manuscript. Addressing the flow of the introduction and trimming repetitive results would greatly improve readability. A more critical reflection on methodology limitations (especially relating to array coverage and tag detection variability) is also recommended.

Major Comments:

- While the introduction contains relevant background information and appropriate citations, it suffers from issues with structure and flow. Several paragraphs appear fragmented or incomplete, and the transitions between key themes (such as ontogeny, conservation, and nursery habitat use) feel abrupt. The authors should consider reorganizing the introduction into a more streamlined narrative, potentially by combining certain paragraphs and ensuring each one clearly builds on the previous in a flowing manner.

**Response: Introduction has been restructured to be more streamlined as requested.**

- Additionally, the introduction frequently uses phrases like “has been documented” without clearly elaborating on the nature or significance of those findings. Rather than simply stating that movement patterns or nursery use have been previously reported, the authors should use this space to immediately and briefly explain how they were documented (e.g., via satellite telemetry, mark-recapture, etc.) and highlight what implications or gaps remain.

**Response: Added how documented and the implications and gaps that remain.**

- A clearer comparison with existing literature (e.g., Gallagher et al. 2021, Hammerschlag et al. 2022) is needed to inform the reader in more depth and emphasize what is new in this paper beyond sample size.

**Response: More in-depth comparison added and what is new about this paper ontogenetic comparison not previously studied in the area.**

- The final paragraph of the Introduction lacks the structure typical of hypothesis-driven studies. It transitions too quickly into a summary of the methods and findings, rather than clearly articulating the study's research objectives and hypotheses. Currently the only value of this study beyond others that I can see is the larger sample size, which typically does not warrant an additional study.

**Response: The larger sample size within each different life-stage of tiger sharks enabled us to compare movements across ontogeny not previously investigated in the area – we have emphasized this now in the introduction.**

- The results section is very long and at times too descriptive. Consider streamlining if possible.

**Response: Results, cutdown reordered and more streamlined.**

- The discussion is thoughtful and highlights ecological mechanisms, but it would benefit from stronger synthesis and focus on key findings to drive the value of this present study.

**Response: Key findings made clearer**

- The finding that sub-adults had highest edge densities is intriguing and consistent with exploratory behaviors during this life-stage. However, the inverse relationship with adults requires more discussion, especially in light of potential detection biases (e.g., seagrass areas with many receivers may inflate local detections).

**Response:** The inverse relationship is discussed. Sub-adults were not highly concentrated in this area and adults were not.

- The GAMs seem appropriate but need further clarity; how were variables selected, and were interactions tested?

**Response: We reran models with sex and FL as an interaction as we realise that this makes sense. The outcome showed a best-fitted model with male FL showing a significant linear effect with edfe density and female FL having a significant non-linear effect (medium sized sharks with the highest edge densities).**

- The issue of shark growth over the study period is acknowledged, but the way this was addressed seems vague. Did the reclassification of life stages affect the statistical models?

**Response: We checked from FL at detection instead of at tagging that’s how we reclassified the five sub-adult individuals to adults. This has been put in Data Analysis and included in the Supporting information table.**

- There are numerous typos, grammatical issues, and awkward phrasings (e.g., “and connectivity was low”, “most were between...”). A thorough review is needed before publication.

**Response: Reviewed and corrected**.

Minor Comments and Edits:

- Title: Consider rephrasing to make it more concise and active, e.g., “Ontogenetic Shifts in Space Use and Habitat Selection of Tiger Sharks in The Bahamas”

**Response: Yes much better – changed.**

- It appears the figure captions are duplicated in the text and at the end with the figures.

**Response:** **We weren’t sure if the captions were only supposed to be in text.**

- The manuscript refers to “Figure 6A,” which implies the existence of additional subpanels (e.g., 6B, 6C), but no such panels are presented or described.

**Response: Correct now an additional subpanel added.**

- Good to see animal welfare and permitting addressed. However, consider briefly summarizing how long handling typically lasted and tagging success rate.

**Response: By tagging success do you mean how many survived? We cannot say but 39 of 46 were detected again others we cannot say where they are.**

- As mentioned in the text, The Bahamas is an expansive archipelago. More attention should be paid to what areas in The Bahamas are being studied and why.

**Response: Two central accessible islands to be able to study shark movements with differ habitat types.**

- Include the sample size and key finding about sub-adults’ motility.
**Response: Included**
- Line 35-40: Too many citations in a row. Add context or split sentences.
**Response: Changed**
- Line 60: Protected from what?
**Response: Fishing – but removed.**
- Line 252: “Gravid implications” is likely a typo for “grave implications”.
**Response: Changed.**
- Line 275: “female tiger sharks are also known to aggregate at Tiger Beach” → include GPS range or region coordinates if possible.
**Response: Added coordinates.**
- Wordy expressions should be revised for clarity. Examples:
- Line 20: “acoustically detected at receiver stations” → simplify to “detected at receiver stations”
- Line 263: "sharks could also be selecting warm shallow seagrass to accelerate growth" → "sharks may use warm shallow seagrass to enhance growth rates"
- Line 275: “high concentration of movements within the same four adjacent shallow seagrass stations” → “highly localized movements among four neighboring seagrass-stations”

**Response: Changed.**

Reviewer #2: Line 76 - What are your specific Aims of the study? You state what you did above, but not what you are trying to resolve/address by this research. This will give your Discussion more direction. Has a network analyses been done before on tiger sharks? What can it tell us that's novel? What are the challenges with this approach for a highly mobile species like this?

**Response: Good points – added to introduction about previous network analysis and this paper is novel because it compares fine scale space use and habitat selection across each life-stage that has not been done before.**

Line 92 - Were sharks also placed in tonic immobility? Was any anesthesia used?

**Response: Yes tonic immobility we have referenced the method used (following Gallagher et al. (2021).**

Line 94 - lower case s for sub-adult
**Response: Changed**

Line 128 - Table S2 needs to include an 'Island' column, so the reader can understand which array they were caught and tagged in.

**Response: Added**
Figure 2 - Suggest as it's not a significant result, move to supp material.

**Response: Removed**
Line 160 - Check your use of 'Adult' vs 'Adults' throughout, currently inconsistent.

**Response:** **Edited**
Line 164 - 14 not fourteen (usually <10 is spelt out - disregard if this is a journal formatting rule).
**Response: Changed**

174 - Were any tagged sharks from one island detected in the other over the duration of the study? This wasn't clear. If so/not, warrants some points in the Discussion.

**Response: Yes – some evidence of connective movements by nine sharks but few – added and made clearer.**

254 - These different habitat types would be good to be captured on a map for readers.

**Response: Added to supporting information.**

264 - Reference needed.

**Response: Added**
274 - Reference needed.

**Response: Added**
278 - From Table S2 I can only assume (until Island is included as a column) that only one mature male was tagged in the New Providence array? Therefore, it's a bit of a stretch to state that only large females were detected here if hardly any mature males were tagged? I think the language here can be softened considerably on these points. Same with using the term 'aggregation' - probably needs some qualification of how you are classifying an aggregation herein. As tiger sharks are semi-solitary, is an 'aggregation' different to what we perceive it to be in a more schooling elasmobranch species?
**Response: Agree the term aggregation removed and we have**

282 - 'Concentration', or regular detection of large individuals moving through this section of the array? Watch for word choice again here.

**Response: Removed concentration.**

284 - Small ones can 'chase' them too - they may not be as successful though. Suggest slight re-word.

**Response: Removed.**

306 - These last two paragraphs are overly focused on prey/diet drivers of habitat use. While important, there is no deep discussion about the habitat types that may be apparent from your detection data by shark size/sex, other possible drivers of space use in your arrays (detections time of day, current strength/direction, tides, other?). Considerations of these ideas may reveal something more important than just prey drivers and will strengthen your Discussion. It may help if you clarify your project Aims clearer above, it will guide your Discussion points better here.

**Response: Main focus was space use and movement between the life stages as Gallagher et al. already focused on looking at drivers of space use and environmental predictors – made this clearer in the intro to not repeat previous study.**

316-328 - Suggest you re-think the points in the paragraph here and their relevance, as I don't recall MPA's being an important part of this study (again, coming back to characterising your Aims/Research Questions for the study earlier should help here).

**Response: Agree MPAS was our focus in Gallagher et al. 2021 - so removed.**

350 - What about outside of The Bahamas? What applicability does the approach you took in this study have to other locations across the world? How is that important for a species like this that connects various regions globally? The Discussion is very parochial in its current form.

**Response: Good point – we have broadened the discussion.**

References - check you ref list for issues like italics for species names etc.

**Response: Checked**

Figure 1 - Needs more details. Most readers won't be from The Bahamas. What islands are each that are zoomed in on? Can they be labelled? Can you also include a larger map of where The Bahamas is in relation to the USA? This is important as you discuss connectivity to the USA.

**Response: Added labels to the map. It already has a lot going on so we don’t think another small map is necessary in addition to the labels.**

Figure 2 - Move to Supp material
**Response: Done**

Overall, this paper has the potential to be a good contribution to the existing literature on tiger sharks once the aforementioned points have been addressed.

6. PLOS authors have the option to publish the peer review history of their article ([what does this mean?](https://na01.safelinks.protection.outlook.com/?url=https%3A%2F%2Ftrack.editorialmanager.com%2FCL0%2Fhttps%3A%252F%252Fjournals.plos.org%252Fplosone%252Fs%252Feditorial-and-peer-review-process%2523loc-peer-review-history%2F1%2F010f01980e13896c-197f2c47-38d3-4968-bb77-203727948264-000000%2FtLmze_EyNBGidgsWd01MvbLh-8lGPyRTw68U8Gq4Oik%3D219&data=05%7C02%7C%7C104aeca9ad1d4b4b992208ddc39bd33d%7C84df9e7fe9f640afb435aaaaaaaaaaaa%7C1%7C0%7C638881796209638047%7CUnknown%7CTWFpbGZsb3d8eyJFbXB0eU1hcGkiOnRydWUsIlYiOiIwLjAuMDAwMCIsIlAiOiJXaW4zMiIsIkFOIjoiTWFpbCIsIldUIjoyfQ%3D%3D%7C0%7C%7C%7C&sdata=V7%2F6EJA0NmjASn8buRe6ubCo9dP4b9aKktX%2FsLH0Ju0%3D&reserved=0)). If published, this will include your full peer review and any attached files.

If you choose “no”, your identity will remain anonymous but your review may still be made public.

**Do you want your identity to be public for this peer review?** For information about this choice, including consent withdrawal, please see our [Privacy Policy](https://na01.safelinks.protection.outlook.com/?url=https%3A%2F%2Ftrack.editorialmanager.com%2FCL0%2Fhttps%3A%252F%252Fwww.plos.org%252Fprivacy-policy%2F1%2F010f01980e13896c-197f2c47-38d3-4968-bb77-203727948264-000000%2F75qQTAYz_Xh0OUkwxHegCXr-D8A4H6WB15MsgowkLEw%3D219&data=05%7C02%7C%7C104aeca9ad1d4b4b992208ddc39bd33d%7C84df9e7fe9f640afb435aaaaaaaaaaaa%7C1%7C0%7C638881796209651183%7CUnknown%7CTWFpbGZsb3d8eyJFbXB0eU1hcGkiOnRydWUsIlYiOiIwLjAuMDAwMCIsIlAiOiJXaW4zMiIsIkFOIjoiTWFpbCIsIldUIjoyfQ%3D%3D%7C0%7C%7C%7C&sdata=Id9vBKa8VNOiu9j6jnT3LvLCUtUYjgIdeqCJFt57a2U%3D&reserved=0).

Reviewer #1: No

Reviewer #2: **Yes:** Bonnie J Holmes

[NOTE: If reviewer comments were submitted as an attachment file, they will be attached to this email and accessible via the submission site. Please log into your account, locate the manuscript record, and check for the action link "View Attachments". If this link does not appear, there are no attachment files.]

While revising your submission, please upload your figure files to the Preflight Analysis and Conversion Engine (PACE) digital diagnostic tool, [https://pacev2.apexcovantage.com/](https://na01.safelinks.protection.outlook.com/?url=https%3A%2F%2Ftrack.editorialmanager.com%2FCL0%2Fhttps%3A%252F%252Fpacev2.apexcovantage.com%252F%2F1%2F010f01980e13896c-197f2c47-38d3-4968-bb77-203727948264-000000%2FXPIfJ_wJjtE7IlDKkwwaRIQOzjZLv2kd6nfSLll8_9k%3D219&data=05%7C02%7C%7C104aeca9ad1d4b4b992208ddc39bd33d%7C84df9e7fe9f640afb435aaaaaaaaaaaa%7C1%7C0%7C638881796209664459%7CUnknown%7CTWFpbGZsb3d8eyJFbXB0eU1hcGkiOnRydWUsIlYiOiIwLjAuMDAwMCIsIlAiOiJXaW4zMiIsIkFOIjoiTWFpbCIsIldUIjoyfQ%3D%3D%7C0%7C%7C%7C&sdata=OdicjyEF9v3RJghOmSv9T0Gv%2FqkDU8iyRskrc8vLxPE%3D&reserved=0). PACE helps ensure that figures meet PLOS requirements. To use PACE, you must first register as a user. Registration is free. Then, login and navigate to the UPLOAD tab, where you will find detailed instructions on how to use the tool. If you encounter any issues or have any questions when using PACE, please email PLOS at [figures@plos.org](mailto:figures@plos.org). Please note that Supporting Information files do not need this step.

*In compliance with data protection regulations, you may request that we remove your personal registration details at any time.* [*(Remove my information/details)*](https://na01.safelinks.protection.outlook.com/?url=https%3A%2F%2Ftrack.editorialmanager.com%2FCL0%2Fhttps%3A%252F%252Fwww.editorialmanager.com%252Fpone%252Flogin.asp%253Fa%3Dr%2F1%2F010f01980e13896c-197f2c47-38d3-4968-bb77-203727948264-000000%2FjLIUMTFableb1d0jvvOyKgb45XdTL2n1Vqh3W-R5IUs%3D219&data=05%7C02%7C%7C104aeca9ad1d4b4b992208ddc39bd33d%7C84df9e7fe9f640afb435aaaaaaaaaaaa%7C1%7C0%7C638881796209679664%7CUnknown%7CTWFpbGZsb3d8eyJFbXB0eU1hcGkiOnRydWUsIlYiOiIwLjAuMDAwMCIsIlAiOiJXaW4zMiIsIkFOIjoiTWFpbCIsIldUIjoyfQ%3D%3D%7C0%7C%7C%7C&sdata=XrbnxGgtEjcaqaiT26rILWZQqu%2B0ZdWuYUSTya8LV88%3D&reserved=0)*. Please contact the publication office if you have any questions.*
