# Supplementary material for: Ontogenetic shifts in space use and habitat selection of tiger sharks (Galeocerdo cuvier) in The Bahamas
Source: PLoS One. 2025 Oct 30;20(10):e0335659. doi: 10.1371/journal.pone.0335659 (PMC12574918; doi:10.1371/journal.pone.0335659)
Supplement: Attachment — Submitted filename: Response to Reviewers.docx [file pone.0335659.s009.docx]

**Response to Reviewers**

Reviewer #1: The authors have made a commendable effort to address the majority of my comments, and the manuscript is much improved in terms of organization, clarity, and methodological detail. However, I feel a few points remain only partially addressed:

- The introduction is better structured, but it still lacks explicit hypotheses. The objectives remain descriptive, and the study would benefit from clearer, testable hypotheses to frame the analyses.

**Response: Hypothesis added**.

- The discussion of adult versus sub-adult edge densities briefly acknowledges potential bias, but the treatment is thin. Differences in receiver density and detection range across habitats (e.g., dense seagrass vs. more open reef or shelf environments) could confound observed patterns, and this is not critically evaluated. More consideration of how array design and habitat-specific detectability may have influenced edge density outcomes would strengthen confidence in the ontogenetic differences reported.

**Response: A new paragraph added discussing this**.

- While the Results have been streamlined, some sections remain overly descriptive, with repetition of percentages and site names that could be condensed.

**Response: Results more condensed.**

- The authors did not include details on handling duration. A brief summary of handling times would improve transparency.

**Response: Range of handling times added.**
